# Numerical Simulation of the Influence of Non-Uniform *ζ* Potential on Interfacial Flow

**DOI:** 10.3390/mi15030419

**Published:** 2024-03-21

**Authors:** Yu Han, Wei Zhao

**Affiliations:** State Key Laboratory of Photon-Technology in Western China Energy, International Collaborative Center on Photoelectric Technology and Nano Functional Materials, Laboratory of Optoelectronic Technology of Shaanxi Province, Institute of Photonics & Photon Technology, Northwest University, Xi’an 710127, China; 202132819@stumail.nwu.edu.cn

**Keywords:** *ζ* potential, electric volume force, microfluidics, solid–liquid interface, streamwise vortex, shear stress

## Abstract

Zeta potential (*ζ* potential) is a significant parameter to characterize the electric property of the electric double layer (EDL), which is important at the solid–liquid interface. Non-uniform *ζ* potential could be developed on a chemically uniform solid–liquid interface due to external flow. However, its influence on the flow has never been concerned. In this investigation, we numerically studied the influence of non-uniform 2D *ζ* potential on the flow at the solid–liquid interface. It is found, that even without any external electric field and only considering the influence of 2D *ζ* potential distribution, swirling flow can be generated near EDL, according to the rotational electric volume force. The streamwise vortices, which are important in the turbulent boundary layer, are theoretically predicted in this laminar flow model when considering the 2D distribution of *ζ* potential, implying the necessity of considering the origin of streamwise vortices of the turbulent boundary layer from the perspective of electrokinetic flow. In addition, the *ζ* potential distribution can promote the wall shear stress. Therefore, more attention must be paid to shear-sensitivity circumstances, like biomedical, medical devices, and in vivo. We hope that the current investigation can help us to better understand the effect of charge distribution on interfacial flow and provide theoretical guidance for the development of related applications in the future.

## 1. Introduction

Electric double layer (EDL) is a kind of physical structure that exists generally in the two-phase interface or even three-phase interface (including solid, liquid, and gas). Taking the solid–liquid interface as an example, when the electrolyte solution flows through the channel, charges will be redistributed on the channel wall. Under the influence of the surface charge, ions in the solution with the opposite charge of the surface charge, i.e., counter-ions, will gather on the surface of the solid, causing the corresponding positive or negative charges to be reordered. Two thin layers with different electric properties can be formed on the interface, including a Stern layer and diffuse layer, constituting the important and common interface electric structure—EDL.

Due to the widespread existence of interfacial phenomena [1,2,3,4,5], EDL and its dynamics play important roles in many fields, such as microfluidic technology [6,7,8], electrochemical analysis [9], energy systems [10,11], and biomedical applications (e.g., pharmaceutical analysis [12] and cell detection [13]). Relying on the electric properties of EDL, the electrokinetic (EK) flow (like electroosmotic flow) under the action of electric field have been broadly applied, to control flow [14], deliver drugs [15], enhance mixing in micro-/nanofluidic devices [16,17], etc. EDL also occupies a central position in solving many global problems (e.g., water desalination [18], sewage treatment [19], etc.).

The Gouy–Chapman–Stern–Grahame (GCSG) model is a widely accepted EDL model, which holds that EDL is composed of a Stern layer and a diffuse layer [20,21,22,23]. The ions in the Stern layer are considered to be adsorbed and fixed on the solid surface, while the ions in the diffuse layer but near the Stern layer cannot move freely due to the strong electrostatic force locally. As the distance from the Stern layer is beyond a certain level, the electrostatic force is not enough to restrain the thermal motion of the ions, and the ions diffuse randomly in the solution, which also shows that the diffuse layer as a dynamic structure can slide on the charged surface. The position where the ions are ready to move is called the shear plane. The *ζ* potential is the potential at the shear plane [23].

The *ζ* potential is a key parameter to characterize the electric structure in EDL. It plays an important role in interface science (e.g., interfacial reaction [24,25], self-assembly [26,27], etc.) and electrokinetic flow dynamics [28,29,30]. However, limited by electrochemical analysis techniques [31,32,33,34] on *ζ* potential, the hypothesis of chemically uniform interfaces with unique *ζ* potential was usually considered in past research. Our understanding of local *ζ* potential and its influence on diverse disciplines is nearly blank.

In recent years, with the in-depth study of nanoscale interface phenomena and the development of new materials with complex surfaces and superstructures, researchers have gradually begun to pay attention to the influence of non-uniform surface charge on the electrical properties of the interface and explore the factors affecting the surface charge distribution and the corresponding potential.

On one hand, relevant research shows that chemically homogeneous slab channel walls exhibit non-uniform *ζ* potential under the action of an outflow field. For example, Lis et al. [35] using vibrational sum frequency generation (v-SFG) spectroscopy preliminarily reveal that surface potentials vary with external flows temporally. Subsequently, Werkhoven et al. [36] showed theoretically that a pressure-driven flow can induce a strong heterogeneous surface charge and *ζ* potential on a chemically homogeneous channel wall. Ober et al. [37] show that liquid flow along the surface of CaF_2_ creates a reversible space charge gradient by v-SFG spectroscopy as well. In a current investigation, relying on a novel fluorescence photobleaching electrochemistry analyzer (FLEA), Meng et al. [38] reported a two-dimensional *ζ* potential induced by external flow at the solid–liquid interface.

On the other hand, non-uniform *ζ* potential can lead to unpredicted outcomes in diverse fields. Paratore et al. [39] demonstrated that the flow that surrounds a surface with non-uniform surface charge density (*ζ* potential as well) can induce complex flow patterns without a physical constraint. Yang et al. [40] revealed that gold superlattices with nanoscale variations in *ζ* potential distribution can significantly augment electrochemical reactions, underscoring the profound impact of *ζ* potential heterogeneity on electrochemical processes.

In the investigation, according to the previous experimental and theoretical reports, we hope to show how the non-uniform *ζ* potential distribution influences the electric volume force and its influence on the flow, through the numerical simulation method.

## 2. Theory

**Figure 1:**
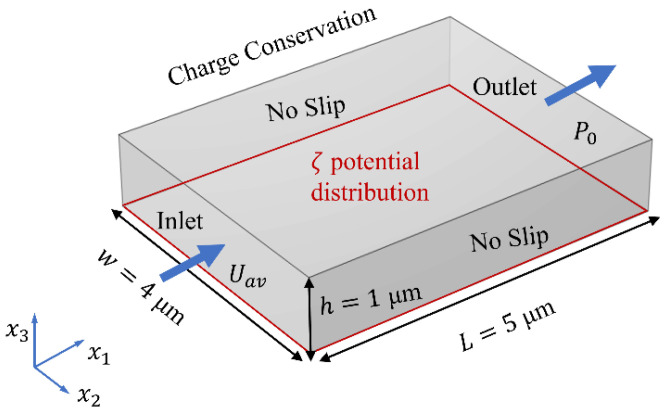
The computational domain, coordinate system, and the boundary conditions of the physical model in the numerical simulation.

The momentum transport process of EK flow can be described by the Navier–Stokes equation with the electric volume force term, which is expressed as:

(1) $$\rho_{f}\left[\frac{\partial \vec{u}}{\partial t}+\left(\vec{u}\cdot \nabla \right)\vec{u}\right]=-\nabla P+\mu \nabla^{2}\vec{u}+\vec{F_{e}}$$

where $\rho_{f}$ is the fluid density, P is the pressure on the fluid, μ is the dynamic viscosity. $\vec{u}=u_{1}\hat{x_{1}}+u_{2}\hat{x_{2}}+u_{3}\hat{x_{3}}$ is the velocity vector, with $u_{1}$, $u_{2}$ and $u_{3}$ being the velocity components in $x_{1}$, $x_{2}$, and $x_{3}$ directions, respectively, and $\hat{x_{1}}$, $\hat{x_{2}}$ and $\hat{x_{3}}$ the corresponding unit vectors in Cartesian coordinates (see Figure 1). $\vec{F_{e}}$ is the electric volume force (EVF), which can be expressed as [41,42,43].

(2) $$\vec{F_{e}}=\rho_{e}\vec{E}-\frac{1}{2}\left(\vec{E}\cdot \vec{E}\right)\nabla \varepsilon +\frac{1}{2}\nabla \left[\rho \vec{E}\cdot \vec{E}{\left(\frac{\partial \varepsilon }{\partial \rho_{f}}\right)}_{T}\right]$$

where $\rho_{e}$ is electric charge density, $\vec{E}$ is the vector of electric field strength. In homogeneous dielectric media with incompressible approximation, the variation of ε is ignored, thus $\vec{F_{e}}=\rho_{e}\vec{E}$, with $\rho_{e}$ and $\vec{E}$ being:

(3) $$\rho_{e}=-\varepsilon \nabla^{2}\varphi$$

(4) $$\vec{E}=-\nabla \varphi$$

where φ is the electric potential in the EDL, $\varepsilon =\varepsilon_{0}\varepsilon_{r}$ is electric permittivity with $\varepsilon_{0}$ being the vacuum permittivity, and $\varepsilon_{r}$ is the relative permittivity of fluid. When the $\zeta =\zeta \left(x_{1},x_{2}\right)$ potential changes slowly, the potential distribution in the EDL can be approximately described by Poisson–Boltzmann theory as:

(5) $$\varphi \left(x_{1},x_{2},x_{3}\right)=\frac{4k_{B}T}{ez_{i}}\tanh^{-1}\left[\tanh\left(\frac{ez_{i}\zeta }{4k_{B}T}\right)exp\left(-\frac{x_{3}}{\lambda }\right)\right]$$

where $k_{B}$ is the Boltzmann constant, T is temperature, e is the elementary charge, and $z_{i}$ is the relative valence state of ions in electrolytes. $\lambda =\sqrt{\varepsilon k_{B}T/2N_{A}e^{2}\sum_{i}c_{i}z_{i}^{2}}$ is the Debye length, which is a characteristic length of the EDL. $N_{A}$ is Avogadro constant, and $c_{i}$ is the concentration of ions in electrolytes.

Under steady state, divide both sides of Equation (1) by $\rho_{f}$, and then calculate the curl on both sides with incompressible fluid relation. Thus,, we have:

(6) $$\left(\vec{u}\cdot \nabla \right)\vec{\omega }-\left(\vec{\omega }\cdot \nabla \right)\vec{u}=\nu \nabla^{2}\vec{\omega }+\vec{T}$$

where $\vec{\omega }=\nabla \times \vec{u}$ is the vorticity, $\nu =\mu /\rho_{f}$ is the kinematic viscosity coefficient, and $\vec{T}=\frac{1}{\rho_{f}}\nabla \times \vec{F_{e}}=T_{1}\hat{x_{1}}+T_{2}\hat{x_{2}}+T_{3}\hat{x_{3}}$ is the driving term of vorticity equation. According to Maxwell’s equations and assuming the magnetic field is steady, by substituting Equations (3) and (4) into $\vec{T}$, we find

(7) $$\vec{T}=\frac{1}{\rho_{f}}\nabla \rho_{e}\times \vec{E}=\frac{\varepsilon }{\rho_{f}}\nabla^{2}\left(\nabla \varphi \right)\times \nabla \varphi$$

For convenience, let $\zeta_{i}=\partial \zeta /\partial x_{i}$, $\zeta_{ij}=\partial^{2}\zeta /\partial x_{i}\partial x_{j}$, $\zeta_{ijk}=\partial^{3}\zeta /\partial x_{i}\partial x_{j}\partial x_{k}$ (with i,j,k are either 1 or 2), from Equation (5), it is obtained

(8) $$\nabla \varphi =\frac{BN\zeta_{1}}{M}\hat{x_{1}}+\frac{BN\zeta_{2}}{M}\hat{x_{2}}+\frac{\psi AB}{\lambda M}\hat{x_{3}}$$

(9) $$\begin{array}{c} \nabla^{2}\varphi =\nabla \cdot \left(\nabla \varphi \right)=\frac{BN\zeta_{11}}{M}-\frac{2ABN\zeta_{1}^{2}}{\psi M}+\frac{2AB^{3}N^{2}\zeta_{1}^{2}}{\psi M^{2}}+\frac{BN\zeta_{22}}{M}-\frac{2ABN\zeta_{2}^{2}}{\psi M}\\ +\frac{2AB^{3}N^{2}\zeta_{2}^{2}}{\psi M^{2}}+\frac{2\psi A^{3}B^{3}}{\lambda^{2}M^{2}}-\frac{\psi AB}{\lambda^{2}M} \end{array}$$

and

(10) $$\begin{array}{cc} \nabla^{2}\left(\nabla \varphi \right)=\nabla \cdot (\nabla^{2} & \varphi )\\ & =\{\frac{BN\zeta_{111}}{M}+\frac{2BN^{2}\zeta_{1}^{3}}{\psi^{2}M}+\frac{4A^{2}BN\zeta_{1}^{3}}{\psi^{2}M}-\frac{6ABN\zeta_{11}\zeta_{1}}{\psi M}+\frac{6AB^{3}N^{2}\zeta_{11}\zeta_{1}}{\psi M^{2}}\\ & -\frac{B^{3}N^{3}\zeta_{1}^{3}}{M^{2}}-\frac{12A^{2}B^{3}N^{2}\zeta_{1}^{3}}{\psi^{2}M^{2}}+\frac{4ABN\zeta_{2}\zeta_{12}}{\psi M}+\frac{4A^{2}BN\zeta_{1}\zeta_{2}^{2}}{\psi^{2}M}+\frac{2AB^{3}N^{2}\zeta_{1}\zeta_{22}}{\psi M^{2}}\\ & +\frac{4AB^{3}N^{2}\zeta_{2}\zeta_{12}}{\psi M^{2}}+\frac{BN\zeta_{1}}{\lambda^{2}M}-\frac{12A^{2}B^{3}N^{2}\zeta_{1}\zeta_{2}^{2}}{\psi^{2}M^{2}}+\frac{8A^{2}B^{5}N^{3}\zeta_{1}\zeta_{2}^{2}}{\psi^{2}M^{3}}\\ & -\frac{8A^{2}B^{3}N\zeta_{1}}{\lambda^{2}M^{2}}+\frac{8A^{4}B^{5}N\zeta_{1}}{\lambda^{2}M^{3}}\}\hat{x_{1}}\\ & +\{\frac{BN\zeta_{222}}{M}+\frac{2BN^{2}\zeta_{2}^{3}}{\psi^{2}M}+\frac{4A^{2}BN\zeta_{2}^{3}}{\psi^{2}M}-\frac{6ABN\zeta_{22}\zeta_{2}}{\psi M}+\frac{6AB^{3}N^{2}\zeta_{22}\zeta_{2}}{\psi M^{2}}\\ & -\frac{B^{3}N^{3}\zeta_{2}^{3}}{M^{2}}-\frac{12A^{2}B^{3}N^{2}\zeta_{2}^{3}}{\psi^{2}M^{2}}+\frac{8A^{2}B^{5}N^{3}\zeta_{2}^{3}}{\psi^{2}M^{3}}+\frac{2BN^{2}\zeta_{2}\zeta_{1}^{2}}{\psi^{2}M}-\frac{2B^{3}N^{3}\zeta_{2}\zeta_{1}^{2}}{\psi^{2}M^{2}}\\ & -\frac{2ABN\zeta_{2}\zeta_{11}}{\psi M}-\frac{4ABN\zeta_{1}\zeta_{12}}{\psi M}+\frac{4A^{2}BN\zeta_{2}\zeta_{1}^{2}}{\psi^{2}M}+\frac{2AB^{3}N^{2}\zeta_{2}\zeta_{11}}{\psi M^{2}}\\ & +\frac{4AB^{3}N^{2}\zeta_{1}\zeta_{12}}{\psi M^{2}}+\frac{BN\zeta_{2}}{\lambda^{2}M}-\frac{12A^{2}B^{3}N^{2}\zeta_{2}\zeta_{1}^{2}}{\psi^{2}M^{2}}+\frac{8A^{2}B^{5}N^{3}\zeta_{2}\zeta_{1}^{2}}{\psi^{2}M^{3}}\\ & -\frac{8A^{2}B^{3}N\zeta_{2}}{\lambda^{2}M^{2}}+\frac{8A^{4}B^{5}N\zeta_{2}}{\lambda^{2}{\left.M\right.}^{3}}\}\hat{x_{2}}\\ & +\{\frac{2A^{2}B^{3}N\zeta_{11}}{\lambda M^{2}}-\frac{BN\zeta_{11}}{\lambda M}-\frac{6AB^{3}N^{2}\zeta_{1}^{2}}{\psi \lambda M^{2}}-\frac{4A^{3}B^{3}N\zeta_{1}^{2}}{\psi \lambda M^{2}}+\frac{8A^{3}B^{5}N^{2}\zeta_{1}^{2}}{\psi \lambda M^{3}}\\ & +\frac{2ABN\zeta_{1}^{2}}{\psi \lambda M}+\frac{2A^{2}B^{3}N\zeta_{22}}{\lambda M^{2}}-\frac{BN\zeta_{22}}{\lambda M}-\frac{6AB^{3}N^{2}\zeta_{2}^{2}}{\psi \lambda M^{2}}-\frac{4A^{3}B^{3}N\zeta_{2}^{2}}{\psi \lambda M^{2}}\\ & +\frac{8A^{3}B^{5}N^{2}\zeta_{2}^{2}}{\psi \lambda M^{3}}+\frac{2ABN\zeta_{2}^{2}}{\psi \lambda M}+\frac{\psi AB}{\lambda^{3}M}-\frac{8\psi A^{3}B^{3}}{\lambda^{3}M^{2}}+\frac{8\psi A^{5}B^{5}}{\lambda^{3}M^{3}}\}\hat{x_{3}} \end{array}$$

where $A\left(x_{1},x_{2}\right)=\tanh\left[\zeta \left(x_{1},x_{2}\right)/\psi \right]$, $B\left(x_{3}\right)=\exp\left(-x_{3}/\lambda \right)$, $M=A^{2}B^{2}-1$, $N=A^{2}-1$, and $\psi =4k_{B}T/ez_{i}$ is thermal potential. Furthermore, after substituting Equations (8)–(10) into $\vec{F_{e}}$ and $\vec{T}$, we have

(11) $$\vec{F_{e}}=\varepsilon \nabla^{2}\varphi \nabla \varphi =(\frac{\varepsilon B^{2}}{M^{2}}N\zeta_{11}-\frac{2AN\zeta_{1}^{2}}{\psi }+\frac{2AB^{2}N^{2}\zeta_{1}^{2}}{\psi M}+N\zeta_{22}-\frac{2AN\zeta_{2}^{2}}{\psi }+\frac{2AB^{2}N^{2}\zeta_{2}^{2}}{\psi M}\;+\frac{2\psi A^{3}B^{2}}{\lambda^{2}M}-\frac{\psi A}{\lambda^{2}})\left(N\zeta_{1}\hat{x_{1}}+N\zeta_{2}\hat{x_{2}}+\frac{\psi A}{\lambda }\hat{x_{3}}\right)$$

and

(12) $$\begin{array}{cc} \vec{T}=\frac{\varepsilon }{\rho_{f}}\nabla^{2}\left(\nabla \varphi \right) & \times \;\nabla \varphi \\ & =\frac{\varepsilon }{\rho_{f}}\frac{B^{2}N}{\lambda M^{2}}\{\left(A^{2}+\frac{B^{2}N^{2}}{M}-\frac{2A^{2}B^{2}N}{M}\right)\zeta_{2}\left(\zeta_{1}^{2}+\zeta_{2}^{2}\right)\\ & +[\left(N\right.+\frac{4A^{2}B^{2}N}{M}-\left.6A^{2}\right)\zeta_{2}\zeta_{22}+\left(N\right.-\left.2A^{2}\right)\zeta_{2}\zeta_{11}\\ & +4A^{2}\left(\frac{B^{2}N}{M}-1\right)\zeta_{1}\zeta_{12}]+\psi A\left(\zeta_{112}{+\zeta }_{222}\right)\}\hat{x_{1}}\\ & -\frac{\varepsilon }{\rho_{f}}\frac{B^{2}N}{\lambda M^{2}}\{\frac{4A}{\psi }\left(A^{2}+\frac{B^{2}N^{2}}{M}-\frac{2A^{2}B^{2}N}{M}\right)\zeta_{1}\left(\zeta_{2}^{2}+\zeta_{1}^{2}\right)\\ & +[\left(N\right.+\frac{4A^{2}B^{2}N}{M}-\left.6A^{2}\right)\zeta_{1}\zeta_{11}+\left(N\right.-\left.2A^{2}\right)\zeta_{1}\zeta_{22}\\ & +4A^{2}\left(\frac{B^{2}N}{M}-1\right)\zeta_{2}\zeta_{12}]+\psi A\left(\zeta_{122}{+\zeta }_{111}\right)\}\hat{x_{2}}\\ & +\frac{\varepsilon }{\rho_{f}}\frac{B^{2}N^{2}}{M^{2}}\{\frac{4A}{\psi }\left(\frac{B^{2}N}{M}-1\right)\left[\zeta_{12}\left(\zeta_{2}^{2}-\zeta_{1}^{2}\right)+\zeta_{1}\zeta_{2}\left(\zeta_{11}-\zeta_{22}\right)\right]\\ & +\left[\zeta_{2}\left(\zeta_{111}+\zeta_{122}\right)-\zeta_{1}\left(\zeta_{112}+\zeta_{222}\right)\right]\}\hat{x_{3}} \end{array}$$

It can be seen when ζ is non-uniform in 2D, $\zeta_{i}$, $\zeta_{ij}$, and $\zeta_{ijk}$ can be non-zero. All the three components in Equation (12) can be nonzero, and thus, the driving term of vorticity is:

(13) $$\vec{T}\neq 0$$

Therefore, based on Equation (6), the electric volume force $\vec{F_{e}}$ with curl can drive the fluid to produce a swirling flow. It is worth noting that, since there is no external electric field applied, we only consider the Coulomb force in Equation (1). The influence of other electric volume forces, e.g., according to electrothermal or dielectric effect, are believed to be absent in this investigation.

## 3. Numerical Simulation

Based on the theory above, we can make a reasonable prediction that in the steady-state flow field, such as pressure-driven flow, there may be a non-uniform two-dimensional *ζ* potential distribution at the solid–liquid interface, which will induce the generation of local electrostatic charge and EVF, resulting in a swirling flow near the interface. To validate this conjecture, we established a mathematical model of non-uniform *ζ* potential distribution and proceeded with a numerical simulation by Comsol Multiphysics on Equations (1)–(5). The influence of non-uniform *ζ* potential distribution at the solid–liquid interface on the flow is investigated under steady-state approximation with the value of relative tolerance is $1\times 10^{-6}$.

### 3.1. Pressure-Driven Laminar Flow

In this simulation model, the material is liquid water, with electric conductivity and pH values are σ=100 μS/cm and pH=9, respectively. The thickness of the EDL on the bottom of the microchannel, i.e., Debye length, is estimated to be 11 nm. The specific physical parameters are shown in Table 1.

The basic flow is a pressure-driven steady flow with incompressible conditions. The bulk flow Reynolds number $R_{e}=Ud/\nu <1$, indicating the basic flow is a typical laminar flow. Here, $d=A/2\left(w+h\right)$ is the hydraulic diameter of the flow, U=Q/A is the bulk flow velocity, Q is flow rate, and A is the cross-sectional area of the computational regime.

In the laminar flow interface, the two sides of the rectangular model that are perpendicular to the $x_{1}$ direction are set to the inlet and outlet, respectively. At the entrance, the fully developed flow condition has been applied, with the average velocity $U_{av}=6.78\times 10^{-5}$ m/s. At the exit, the boundary condition is suppressing backflow (which adjusts the outlet pressure to reduce the amount of fluid entering the domain through the exit), with static pressure $P_{0}=0$ Pa. According to the symmetry of the geometric model and the rationality of the physical model, a no slip boundary condition has been imposed on the other four walls (including the upper wall, bottom wall, and two side walls), where the fluid velocity is zero. Electric volume force $\vec{F_{e}}$ has been applied to the entire domain of the model (see Equation (5), similar to potential φ, and $\vec{F_{e}}$ is dominant in the EDL at the bottom of microchannel), as shown in Figure 1.

### 3.2. Electrostatics Module

In this investigation, there is no external electric field applied, and the electric volume force $\vec{F_{e}}$ is entirely generated by the 2D distribution of *ζ* potential. To study the effect of the electrical volume force $\vec{F_{e}}$ due to the non-uniform *ζ* potential on the flow field, the electrostatic interface has been added into the simulation model. Furthermore, since the electric field is steady, the effect of the magnetic field is negligible.

The charge is conserved throughout the domain, and the electroosmotic potential φ calculated by Equation (5) was applied in the entire physical model. To further reduce the computational cost, we calculate the electric volume force by defining custom variables and analytic functions according to Equations (2)–(5).

**Figure 2:**
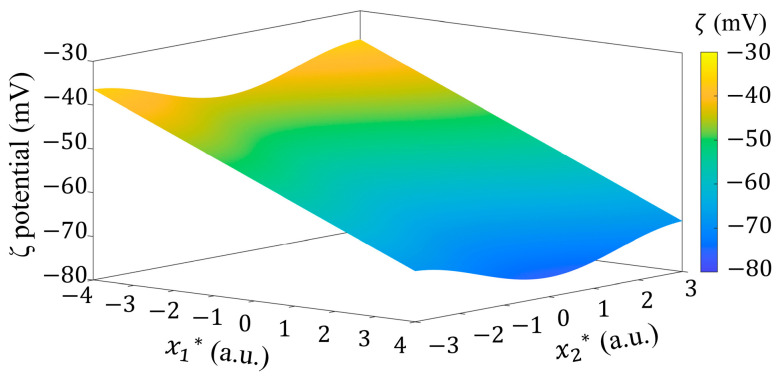
The non-uniform 2D *ζ* potential distribution with k=222 1/m, $A^{*}=0.1$, $\zeta_{0}=-36$ mV and $k_{2}=1/\pi$.

As mentioned above, in order to investigate the effect of non-uniform *ζ* potential distribution on interfacial flow, a mathematical model of *ζ* potential distribution (as plotted in Figure 2) was established to characterize the *ζ* potential distribution in the solid–liquid interface at the bottom of the microchannel only (as shown in Figure 1) based on the recent report of Meng et al. [38], as

(14) $$\zeta \left(x_{1},x_{2}\right)=\left[kx_{1}+A^{*}\cos\left(2\pi k_{2}x_{2}\right)+1\right]\zeta_{0}$$

where $\zeta_{0}$ is the classical *ζ* potential value at the interface of water-fused silica [44]. The slope k represents how quickly the *ζ* potential increases in the streamwise direction (with ζ<0). The dimensionless amplitude $A^{*}$ represents the variation of *ζ* potential along the spanwise direction. The wavenumber $k_{2}$ determines the possible periodicity of *ζ* potential in the spanwise direction.

By substituting Equation (14) into Equation (5), electroosmotic potential φ could be calculated. In turn, the electric charge density $\rho_{e}$, electric field strength $\vec{E}$, EVF, and $\vec{T}$ could be calculated accordingly by Equations (3), (4), (11) and (12).

Finally, it is worth noting that due to the choice of steady-state study method, initial values of velocity, pressure, and potential are not considered when the boundary conditions are set.

### 3.3. Modeling and Meshing

The computational regime is a cuboid with dimensions in the order of micrometers. In the $x_{1}$-$x_{2}$ plane, the geometric model is built around the origin O(0,0). In the investigation, the potential φ (see Equation (5)) decreases exponentially with $x_{3}$ and is dominant only in the EDL at the solid–liquid interface. To capture the influence on flow dynamics, grid cells with nanoscale resolution are required. However, to be able to capture the evolution of flow, a large computational domain is required. To compromise these two requirements, the computation regime is L=5 μm long, w=4 μm wide, and h=1 μm high. Thus, we can capture the influence of micrometer variation of *ζ* potential on the flow. In the modeling process, the geometric model is divided into three regions in the $x_{3}$ direction, including a large bulk region ($h_{b}=0.9$ μm) in the center of the channel and two symmetrical thin-layer regions ($h_{t}=50$ nm) near the bottom and upper wall.

**Figure 3:**
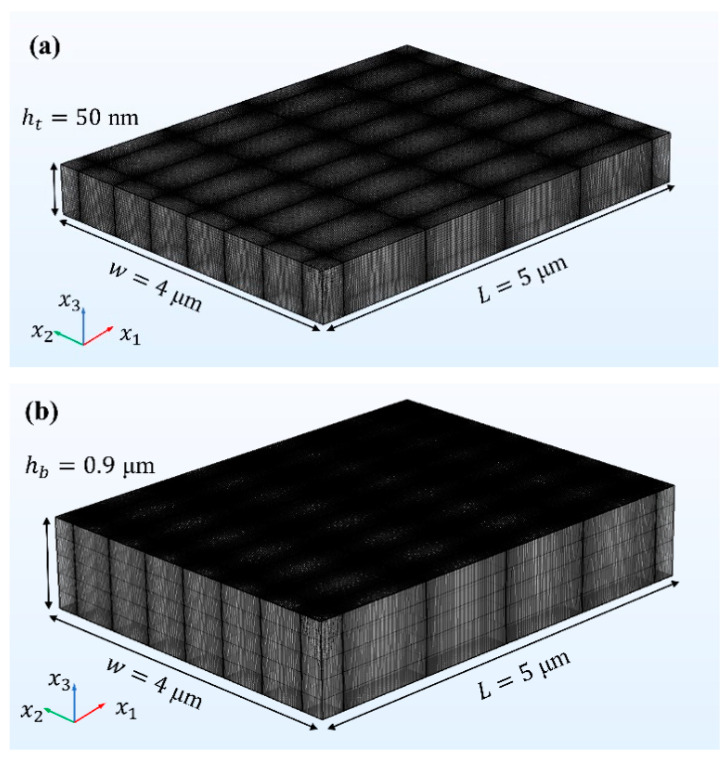
The meshing of solution domain: (**a**) the thin-layer region (the schematic is scaled up to 10 times in the $x_{3}$ direction) and (**b**) the large bulk region.

In the meshing process, mapping operation is used in the $x_{1}$-$x_{2}$ plane, and sweeping operation is carried out upward from the bottom to the upper wall in the $x_{3}$ direction and downward from the ceiling to the under wall in the opposite of the $x_{3}$ direction, respectively, in the two thin-layer regions. The grid can be denser in the target area by the arrangement of geometric sequence. Detailed grid distribution of each region is shown in Figure 3.

During the sweeping operation in the $x_{3}$ direction, the grid in the thin-layer region is divided down to the nanometer scale to ensure resolution. The minimum and maximum grid sizes are 1.33 nm and 2.66 nm, respectively. While in the bulk region, the grid is much coarser with a fixed size of 180 nm. In the $x_{1}$-$x_{2}$ plane, both bulk region and thin-layer region have the same minimum grid size and the same maximum grid size. In the $x_{1}$ direction, the minimum and maximum grid sizes are 6.68 nm and 20 nm, respectively. In the $x_{2}$ direction, the minimum and maximum grid sizes are 4.42 nm and 13.26 nm, respectively. Hexahedral grid elements are used for all regions in the model. Detailed parameters for complete grid statistics of the model are shown in Table 2.

### 3.4. Grid Independence Analysis

**Figure 4:**
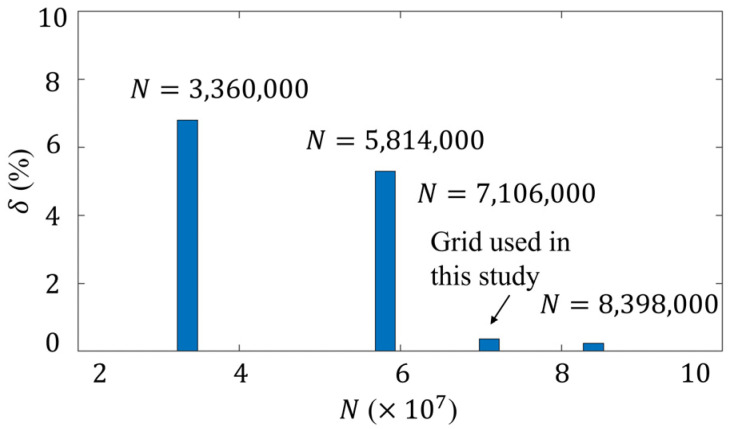
Grid independence analysis. Plot of relative errors δ on the computation of velocity, versus the grid quantity N, with k=55.5 1/m, $A^{*}=0.1$, $\zeta_{0}=-36$ mV and $k_{2}=1/\pi$.

To ensure computational validity and stability, we conducted a grid independence analysis by systematically varying the grid size in all dimensions while keeping other parameters such as boundary conditions and solvers unchanged. The relative errors (δ) of the simulated velocity field between the models of different grid quantities (N) are plotted in Figure 4. It can be seen that δ decreases asymptotically towards zero, with the increase in grid quantity. The minimum value is 0.24%. The results show that when the grid quantity is equal to or beyond N=7,106,000, δ is within 1%, indicating a negligible dependency of computational results on grid density [45]. Consequently, to strike a balance between computational efficiency and precision, we adopted a grid resolution of N=7,106,000 for subsequent analyses in this study.

## 4. Results

### 4.1. Pressure-Driven Basic Flow

In the previous investigations on interfacial flow, the influence of interfacial charge distribution and ζ potential distribution was not taken into account. The basic flow (e.g., common Poiseuille flow) is driven only by pressure.

**Figure 5:**
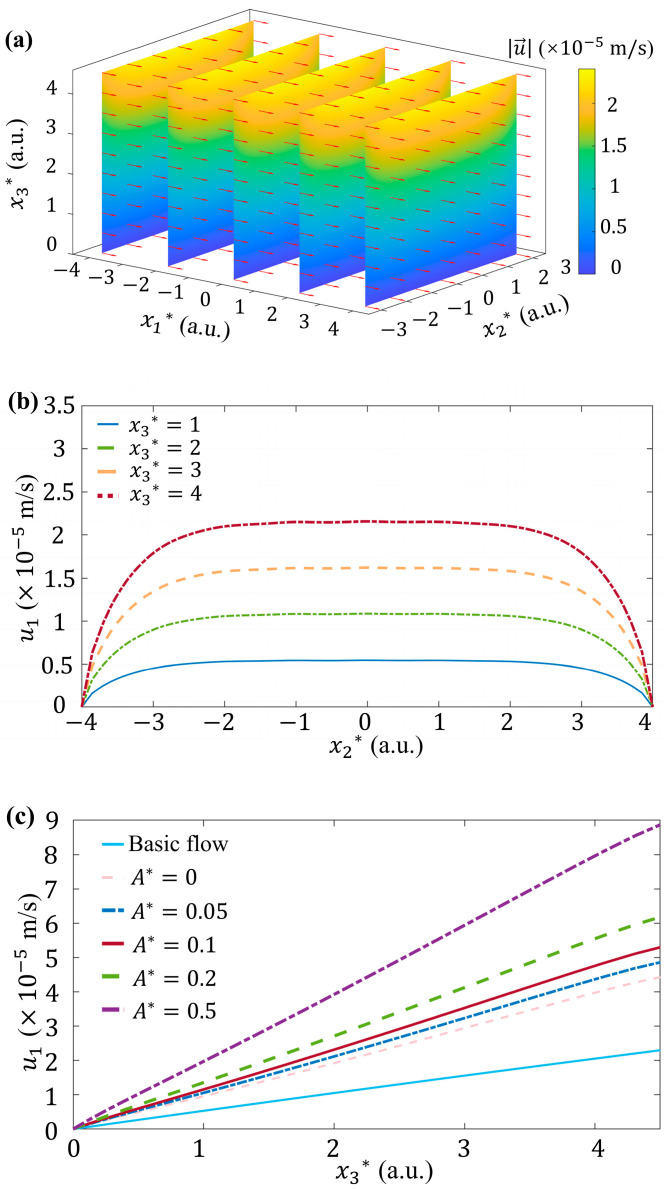
Velocity distributions. (**a**) 3D distribution of fluid velocity $\vec{u}$ of basic flow. The magnitudes of $\vec{u}$, i.e., $\left|\vec{u}\right|$, is demonstrated by color. The direction of $\vec{u}$, evaluated by $\vec{u}/\left|\vec{u}\right|$, is demonstrated by the arrows with unit length. (**b**) Plot of the streamwise velocity component of basic flow, $u_{1}$, versus ${x_{2}}^{*}$ at different ${x_{3}}^{*}$ with ${x_{1}}^{*}=4$. (**c**) Plot of streamwise velocity component, $u_{1}$, versus ${x_{3}}^{*}$ at different $A^{*}$, where ${x_{1}}^{*}=4$ and ${x_{2}}^{*}=0$ with k=222 1/m, $\zeta_{0}=-36$ mV and $k_{2}=1/\pi$.

In this case, the velocity field distribution of the basic flow is shown in Figure 5a, where ${x_{1}}^{*}=x_{1}/l$, ${x_{2}}^{*}=x_{2}/l$, ${x_{3}}^{*}=x_{3}/\lambda$ with characteristic length l=h/2=0.5 μm and Debye length λ=11 nm. We applied a small volume rate (Q=0.001 μL/h), where the basic flow velocity is $1.2\times 10^{-4}$ m/s at 0.5 µm from the wall, to simulate a relatively large wall strain rate (240 s^−1^). Slice and quiver diagrams are used to show the distribution of the intensity (where the magnitude of intensity is represented by the color) and direction of the fluid velocity $\vec{u}$ in the $x_{1}$-$x_{3}$ planes at different streamwise positions. The direction of the fluid velocity after normalization at each position is represented by the vector arrow. Due to the absence of the external force, the velocity of the basic flow shows an approximately parabolic distribution in the spanwise direction near the wall, as can be found in Figure 5b with ${x_{1}}^{*}=4$.

In contrast, as shown in Figure 5c, since the EDL is extremely thin, the streamwise component of velocity, i.e., $u_{1}$, shows a linear variation with the dimensionless vertical position ${x_{3}}^{*}$. This is an approximation of the parabolic velocity distribution of Poiseuille flow in the thin EDL.

### 4.2. Uniform ζ Distribution

**Figure 6:**
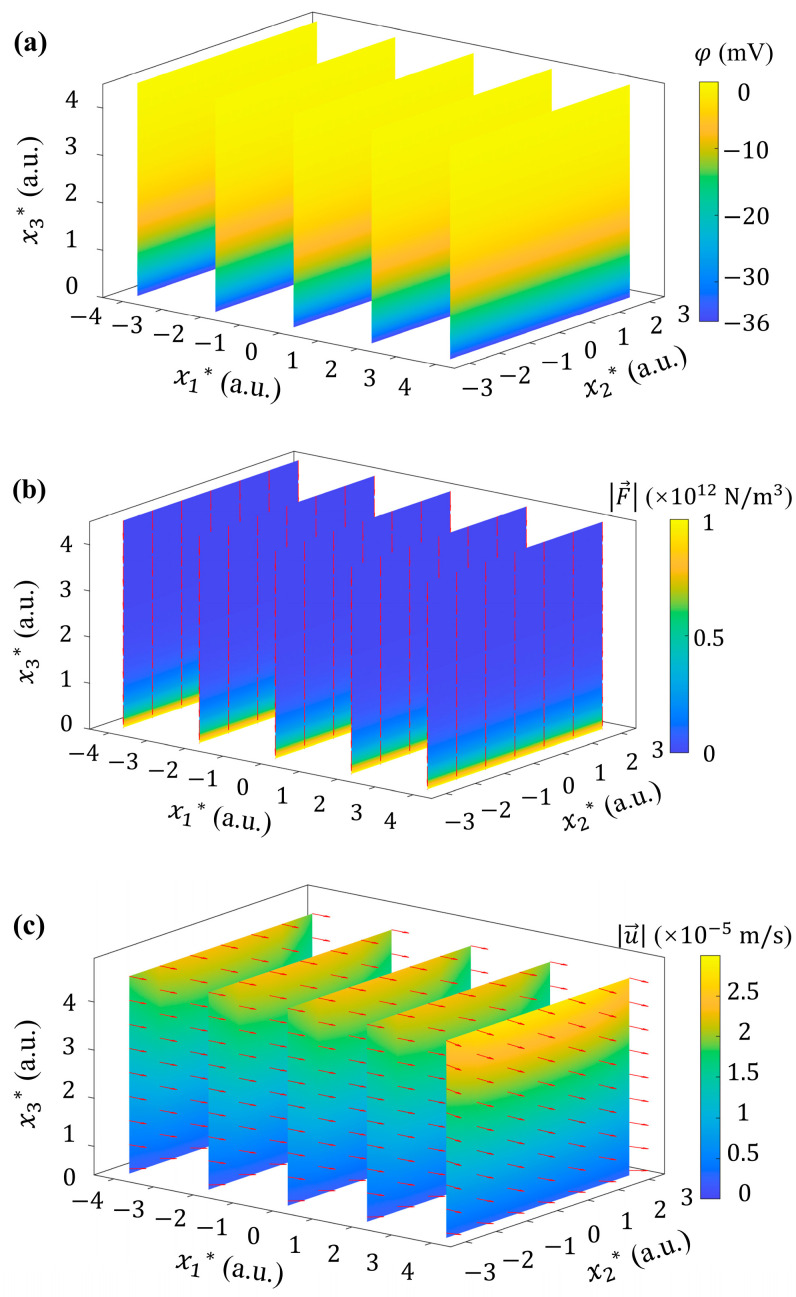
Potential, electric volume force, and velocity fields generated by uniform *ζ* potential with ζ=−36 mV. 3D distribution of (**a**) electroosmotic potential φ, (**b**) electric volume force $\vec{F_{e}}$, and (**c**) fluid velocity $\vec{u}$.

When considering the presence of uniformly distributed *ζ* potential (i.e., $\zeta =\zeta_{0}$) at the solid–liquid interface, based on Equation (5), it can be seen that there is an electroosmotic potential distribution in Figure 6a. It shows a uniform distribution in the $x_{1}$-$x_{2}$ plane and exponential decay in the vertical direction. Accordingly, $\vec{F_{e}}$ are all in the negative $x_{3}$ direction with decreasing magnitudes from bottom to top, as shown in Figure 6b. $\vec{T}$ is exactly zero everywhere. In other words, the uniform distribution of the *ζ* potential induces irrotational $\vec{F_{e}}$. Under the influence of $\vec{F_{e}}$, the velocity distribution of the flow field is shown in Figure 6c. Compared with Figure 5a, it is obvious that the direction of the velocity $\vec{u}$ has not changed, which still dominated by the $x_{1}$-directional component $u_{1}$. Furthermore, the parabolic velocity distribution in the $x_{2}$ and $x_{3}$ directions are still present.

From the perspective of vortex dynamics, the source term $\vec{T}$ in Equation (6) does not exist in either the basic flow or the flow field considering uniform *ζ* potential. Therefore, it does not disturb the basic flow, nor drive the fluid to produce vorticity, and cannot cause the vorticity to deform (stretch, tilt, twist, etc.).

### 4.3. One-Dimensional ζ Distribution

In this subsection, we consider if an 1D distribution of *ζ* potential with a certain slope in the *x* direction is formed due to nonuniform interface charge redistribution by the basic flow. Let $A^{*}=0$ in Equation (14), we have:

(15) $$\zeta \left(x_{1}\right)=\left(kx_{1}+1\right)\zeta_{0}$$

**Figure 7:**
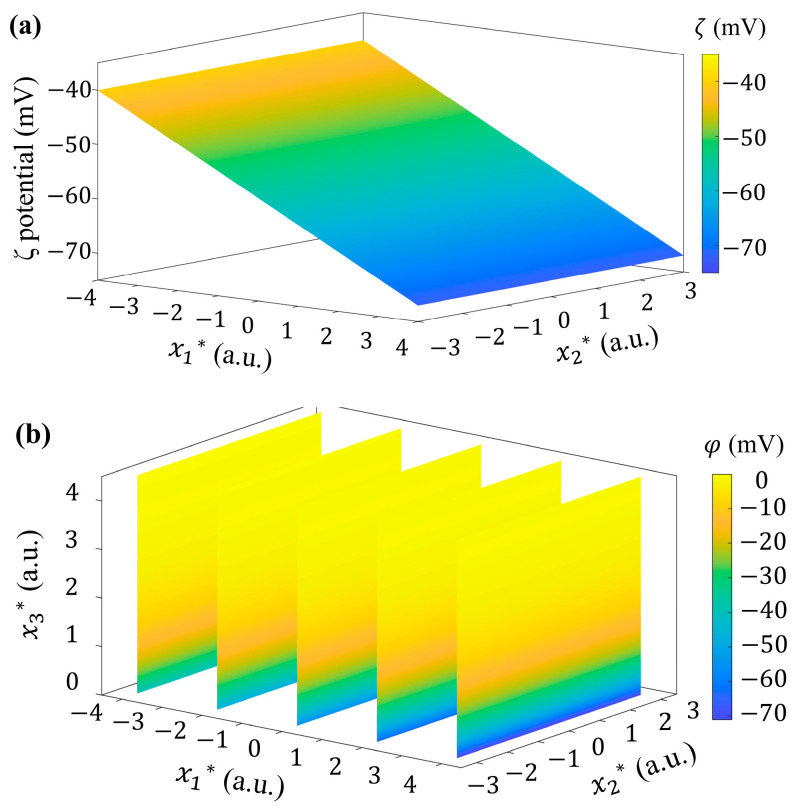

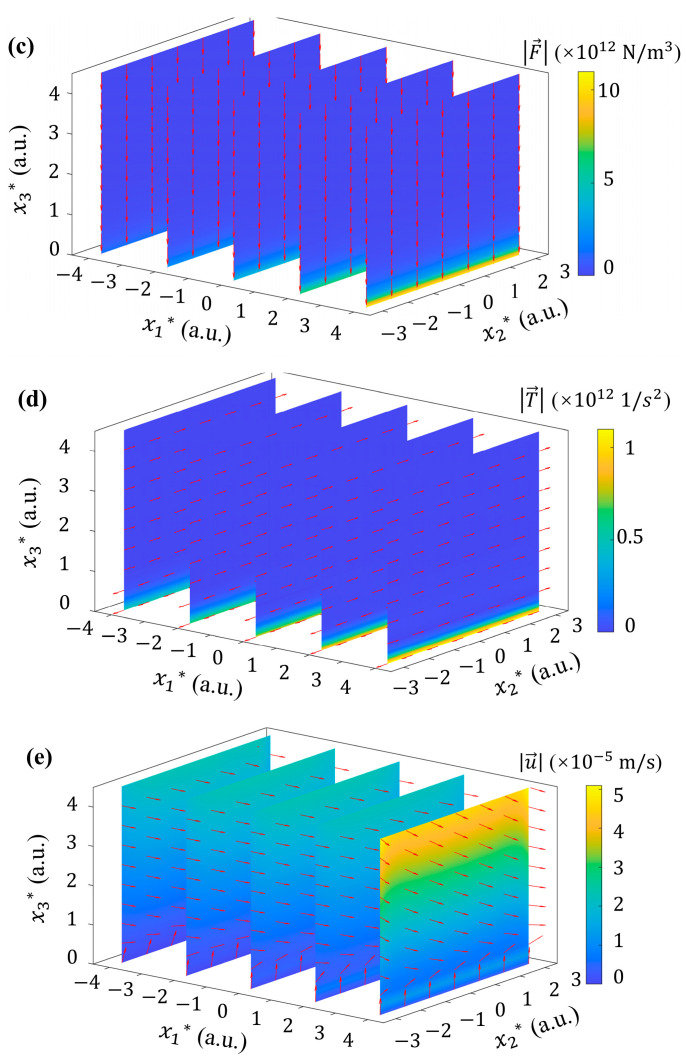
Electric field and flow field generated by 1D *ζ* potential with k=222 1/m and $\zeta_{0}=-36$ mV. (**a**) 1D distribution of *ζ* potential. 3D distribution of (**b**) electroosmotic potential φ, (**c**) electric volume force $\vec{F_{e}}$, (**d**) the curl of electric volume force $\vec{T}$, and (**e**) fluid velocity $\vec{u}$ under this 1D *ζ* potential distribution.

Taking k=222 1/m and $\zeta_{0}=-36$ mV as an example, the 1D distribution of *ζ* potential see Figure 7a, and the distributions of φ and $\vec{F_{e}}$ induced by this *ζ* potential are shown in Figure 7b and Figure 7c, respectively. Based on the increase in $\left|\zeta \right|$ in the $x_{1}$ direction, the strength of $\vec{F_{e}}$ also shows an increasing trend from upstream to downstream. Compared with Figure 6b, although the $x_{3}$ component of $\vec{F_{e}}$ is still dominant, the $x_{1}$ component of the $\vec{F_{e}}$ is not zero anymore. This subsequently means that the $x_{2}$ component of $\vec{T}$, i.e., $T_{2}\neq 0$, as shown in Figure 7c.

Although the flow with 1D *ζ* potential distribution can induce the electric volume force with ${F_{e}}_{1}\neq 0$ and ${F_{e}}_{3}\neq 0$, in this case, we still have the streamwise and vertical components of $\vec{T}$, i.e., $T_{1}=T_{3}=0,$ which could be seen more intuitively in Figure 7d. According to Equation (6), $\omega_{1}=\omega_{3}=0$. There is no vorticity generated in streamwise and vertical directions through the electrical volume force, except for $T_{2}$ and $\omega_{2}$. As a result, the velocity distribution of the flow field in Figure 7e shows a clear difference from Figure 5a and Figure 6c. The dominancy of $u_{1}$ is no longer always true; in contrast, $u_{3}$ becomes dominant when $0<{x_{3}}^{*}<1$. Compared with the basic flow, both $u_{1}$ (Figure 5c) and $\left|\vec{u}\right|$ have exhibited significant increment. The parabolic distribution in the $x_{2}$ direction of basic flow (see Figure 5b) is no longer present when considering 1D *ζ* potential distribution.

### 4.4. Two-Dimensional ζ Distribution

In this subsection, the influence of the 2D distribution of *ζ* potential on flow is investigated. In Equation (14), we take k=222 1/m, $A^{*}=0.1$, $\zeta_{0}=-36$ mV, and $k_{2}=1/\pi$ as an example. The *ζ* potential exhibits a 2D distribution on a solid–fluid surface, as plotted in Figure 2.

**Figure 8:**
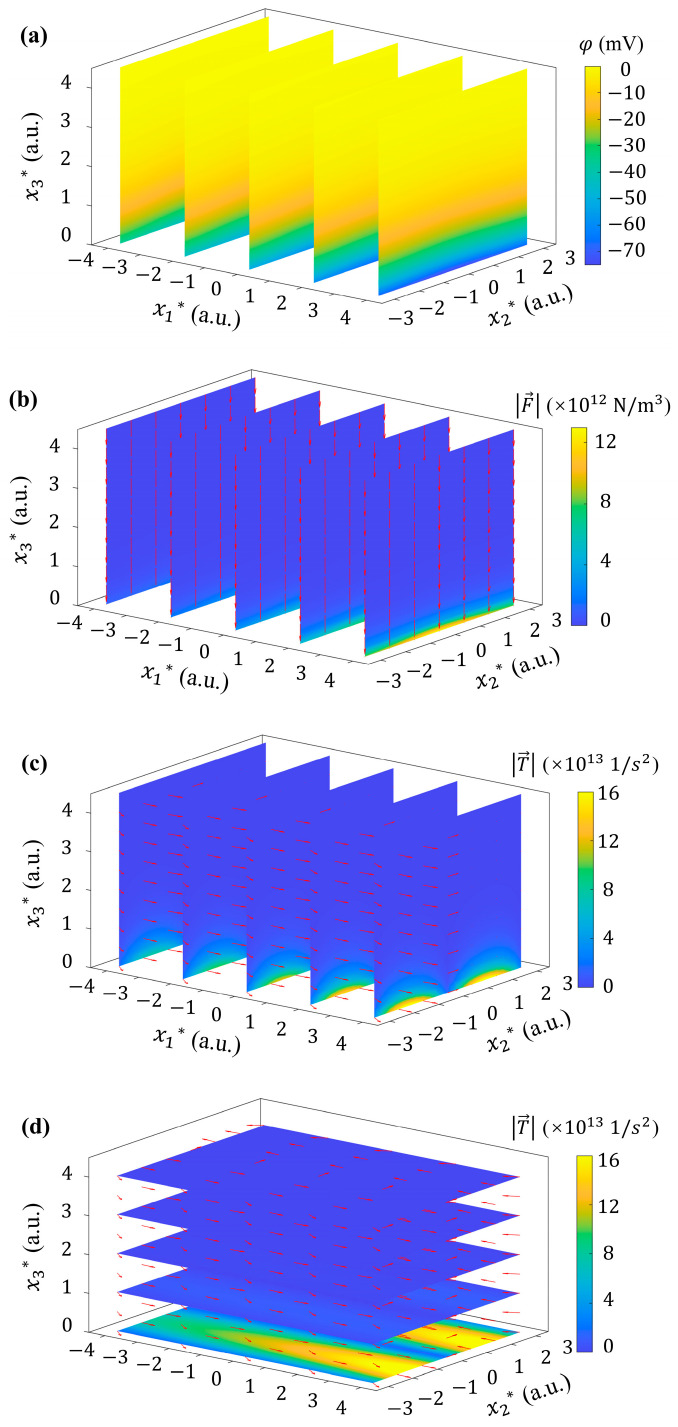
Potential, $\vec{F_{e}}$ and $\vec{T}$ generated by 2D *ζ* potential with k=222 1/m, $A^{*}=0.1$, $\zeta_{0}=-36$ mV and $k_{2}=1/\pi$. (**a**) 3D distribution of φ. (**b**) 3D distribution of electric volume force $\vec{F_{e}}$. (**c**,**d**) 3D distribution of $\vec{T}$.

Based on Equations (2)–(5), a 3D electroosmotic potential φ distribution is generated in the EDL at the bottom of the microchannel (as shown in Figure 8a), which in turn generates local electric charge and induces a non-uniform electric volume force. As shown in Figure 8b, $\vec{F_{e}}$ exhibits strong $x_{3}$-directional component. The directions of $\vec{F_{e}}$ are mainly pointing to the bottom wall of the microchannel. The strength of $\vec{F_{e}}$ generally shows a decaying trend along $x_{3}$, reaching a maximum at one Debye length above the bottom wall. The strength of $\vec{F_{e}}$ also exhibits variations along the streamwise direction and the spanwise direction. It increases gradually downstream and decreases from the center of the flow field, forming an approximate symmetry distribution along the spanwise direction.

Different from the $\vec{F_{e}}$ induced by 0D (Figure 6b) and 1D (Figure 7c) distributions of *ζ* potential, in the model of 2D *ζ* potential, $\vec{F_{e}}$ can be rotational in 3D. All three components of $\vec{T}$ can be non-zero, as shown in Figure 8c,d. $\left|\vec{T}\right|$ increases in the streamwise direction, which is consistent with that of $\left|\vec{F_{e}}\right|$. In the spanwise, $\left|\vec{T}\right|$ shows an approximate symmetry with respect to the center of the channel, but in opposite directions (see Figure 8d). Two maxima can be observed at ${x_{2}}^{*}=$ −1.5 and 1.5.

**Figure 9:**
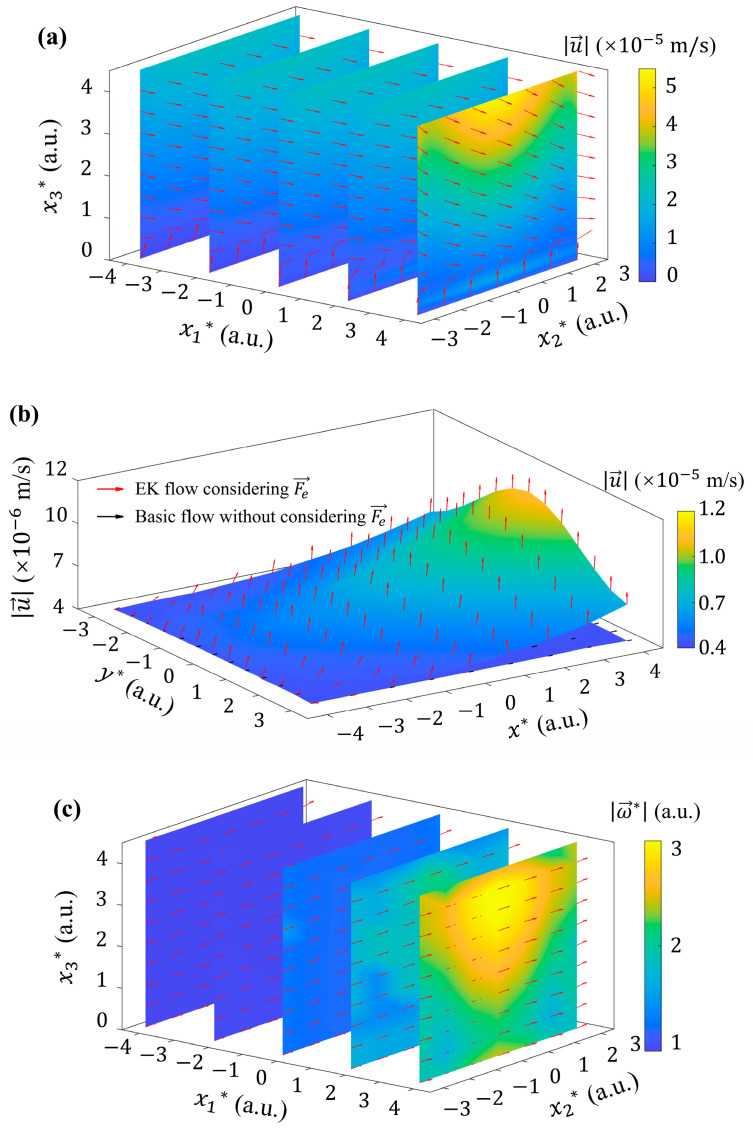
Velocity and vorticity fields generated by 2D *ζ* potential with k=222 1/m, $A^{*}=0.1$, $\zeta_{0}=-36$ mV and $k_{2}=1/\pi$. (**a**) 3D distribution of fluid velocity $\vec{u}$. (**b**) 2D velocity distribution of flow considering and without considering *ζ* potential distribution at ${x_{3}}^{*}=1$. (**c**) 3D distribution of the curl of fluid velocity $\vec{\omega }$, denoted by $\left|{\vec{\omega }}^{*}\right|$, with ${\vec{\omega }}^{*}=\vec{\omega }/\left|{\vec{\omega }}_{ref}\right|$. Here, $\left|{\vec{\omega }}_{ref}\right|$ is the maximum $\left|\vec{\omega }\right|$ of basic flow.

The fluid velocity distribution under the driven of rotational $\vec{F_{e}}$ is shown in Figure 9a. The vertical velocity component $u_{3}$ was observed near the bottom within one Debye length (i.e., $0<{x_{3}}^{*}<1$). While $u_{1}$ component begins to dominate at ${x_{3}}^{*}\geq 1$. This phenomenon suggests that even though most of the electric volume force is offset by pressure gradient, such a large volume force will still have a significant impact on the velocity field. At the height of ${x_{3}}^{*}\approx 2.5$ and higher, $\left|\vec{u}\right|$ is concentrated in the center of the microchannel in the spanwise to reach the maximum, which shows significant difference from those in Figure 5a, Figure 6c and Figure 7e.

In order to further explore the influence of 2D *ζ* potential on the interfacial flow, the velocity distribution in the $x_{1}$-$x_{2}$ plane that one Debye length from the bottom (${x_{3}}^{*}=1$) is shown in Figure 9b by a surface diagram. It can be seen that the velocity $\left|\vec{u}\right|$ of the flow, considering the effect of $\vec{F_{e}}$ caused by 2D *ζ* potential distribution, is about 3 folds higher than the basic flow. It exhibits an obvious increasing trend from upstream to downstream in the streamwise direction. In this plane, $u_{3}$, rather than $u_{1}$, is dominant among the three components of the flow velocity, indicating that the electric volume force (after being mostly balanced by the pressure term of Equation (1)) can cause the development of flow at the bottom wall. This implies, when considering the EK flow driven by 2D *ζ* potential, the momentum transport near the interface can be significantly promoted.

The non-zero $\vec{T}$ further generates $\vec{\omega }$ in the flow field, as shown in Figure 9c. Particularly, the streamwise component of vorticity is non-zero, i.e., $\omega_{1}\neq 0$, and is gradually strengthened from upstream to downstream. In the upstream region, $\vec{\omega }$ mainly aligns along the $x_{2}$ direction with approximately uniform distribution on the same $x_{3}$. While in the downstream, this distribution is broken. $\vec{\omega }$ becomes bending towards the streamwise direction, indicating the possible generation of streamwise vortices.

Streamwise vortices commonly exist in flow boundary layers, e.g., the well-known coherent vortex structures in the turbulent boundary layer. However, the origin of such vortices remains unclear. We hope to give an insight into this problem through the vision of electrokinetic mechanism, and provide a new approach to control the generation and evolution of vortices in boundary layers.

## 5. Discussion

In this section, the influence of the parameters (such as slope k, dimensionless amplitude $A^{*}$, initial zeta potential $\zeta_{0}$, and wavenumber $k_{2}$) on the flow are systematically investigated by numerical simulations.

### 5.1. Influence of k

**Figure 10:**
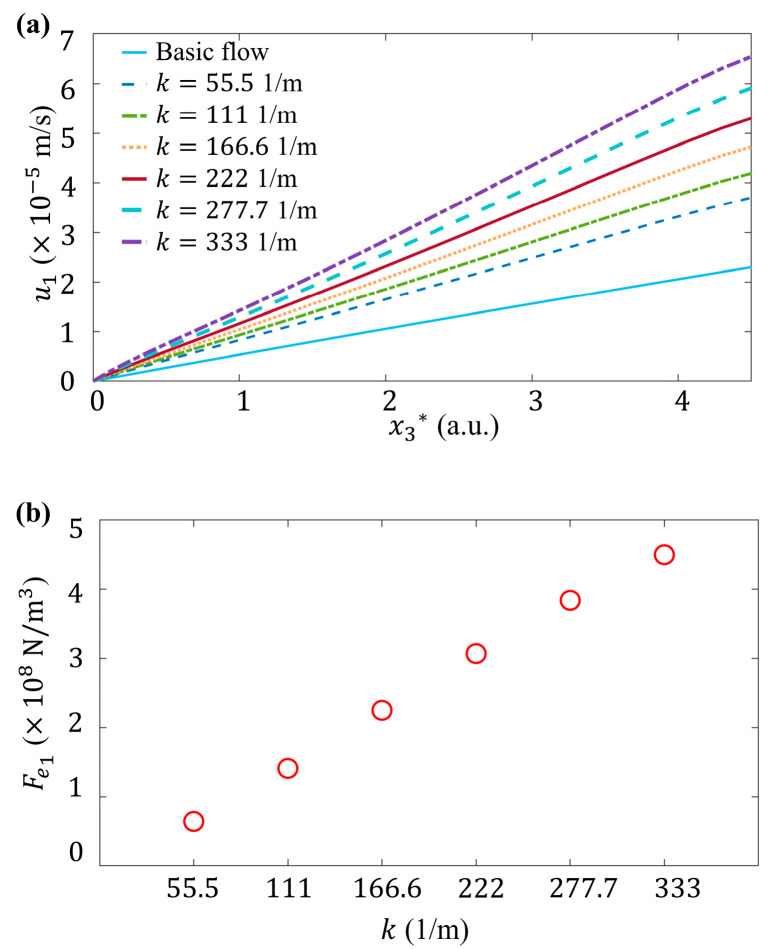

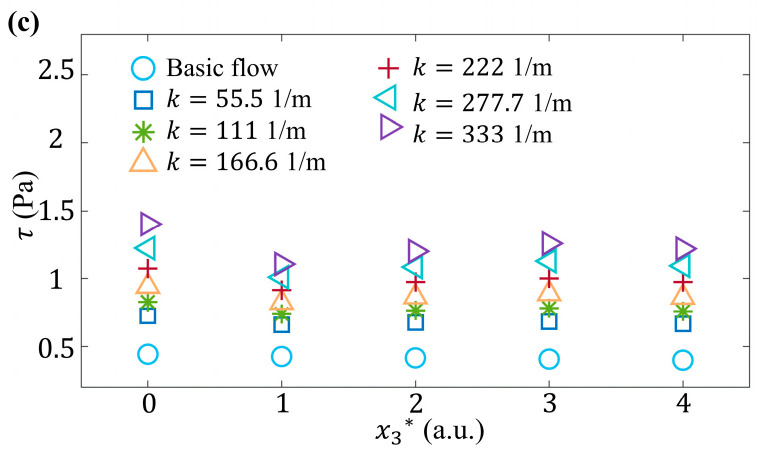
Influence of k on the flow, where ${x_{1}}^{*}=4$ and ${x_{2}}^{*}=0$, $A^{*}=0.1$, $\zeta_{0}=-36$ mV, and $k_{2}=1/\pi$. (**a**) Plot of streamwise velocity component, $u_{1}$, versus dimensional height ${x_{3}}^{*}$ at different k. (**b**) Plot of streamwise electric volume force component ${F_{e}}_{1}$ versus slope k at the height of ${x_{3}}^{*}=1$. (**c**) Plot of wall shear stress, τ, versus dimensional height ${x_{3}}^{*}$ at different k.

In this subsection, the influence of slope k on the flow is investigated at $A^{*}=0.1$, $\zeta_{0}=-36$ mV, and $k_{2}=1/\pi$. In Figure 10a, it is obvious that $u_{1}$ shows a linear increment with ${x_{3}}^{*}$, which is an approximation of the parabolic distribution caused by fluid viscosity in a thin layer. When the slope k gradually increases, a stronger variation of *ζ* potential along the streamwise direction is induced. The streamwise component of electric volume force gradually increases as well (see Figure 10b), accompanied by the increasing $u_{1}$ and velocity gradient $\partial u_{1}/\partial x_{3}$. Thus, the wall shear stress is:

(16) $$\tau =\mu \frac{\partial u_{1}}{\partial x_{3}}$$

which considering 2D *ζ* potential distribution can be significantly stronger than that of basic flow, as can be seen more clearly in Figure 10c.

### 5.2. Influence of $A^{*}$

The influence of $A^{*}$ is investigated at k=222 1/m, $\zeta_{0}=-36$ mV and $k_{2}=1/\pi$, as shown in Figure 5c. The comparison between the basic flow and the flow under 1D *ζ* potential (i.e., $A^{*}=0$) shows that the fluid velocity can be enhanced even if $T_{1}=T_{3}=0$. When spanwise variation of *ζ* is taken into account, both $u_{1}$ and velocity gradient $\partial u_{1}/\partial x_{3}$ are strengthened at larger $A^{*}$, indicating stronger wall shear stress as well.

### 5.3. Influence of $\zeta_{0}$

**Figure 11:**
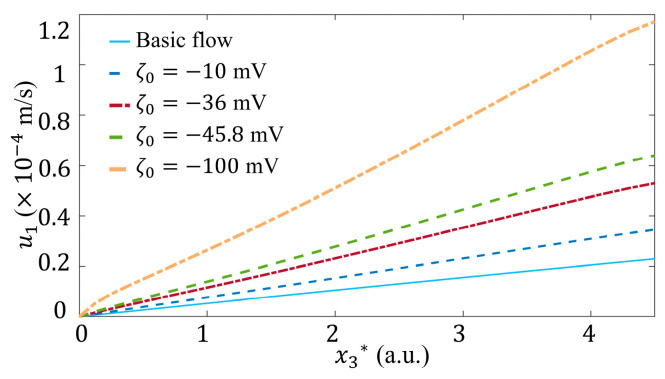
Influence of $\zeta_{0}$ on the flow, where ${x_{1}}^{*}=4$ and ${x_{2}}^{*}=0$, k=222 1/m, $A^{*}=0.1$, and $k_{2}=1/\pi$. Plot of streamwise velocity component, $u_{1}$, versus dimensional height ${x_{3}}^{*}$ at different $\zeta_{0}$.

$\zeta_{0}$ is the *ζ* potential in quiescent fluid. It is a constant in a chemically uniform interface, determined by material properties and irrelevant to the flow conditions. Similar as k and $A^{*}$, as $\zeta_{0}$ is decreased from −10 mV [28], −36 mV [44], −45.8 mV [22], to −100 mV [20], $u_{1}$ (Figure 11) and its gradient $\partial u_{1}/\partial x_{3}$ increase apparently.

### 5.4. Influence of $k_{2}$

**Figure 12:**
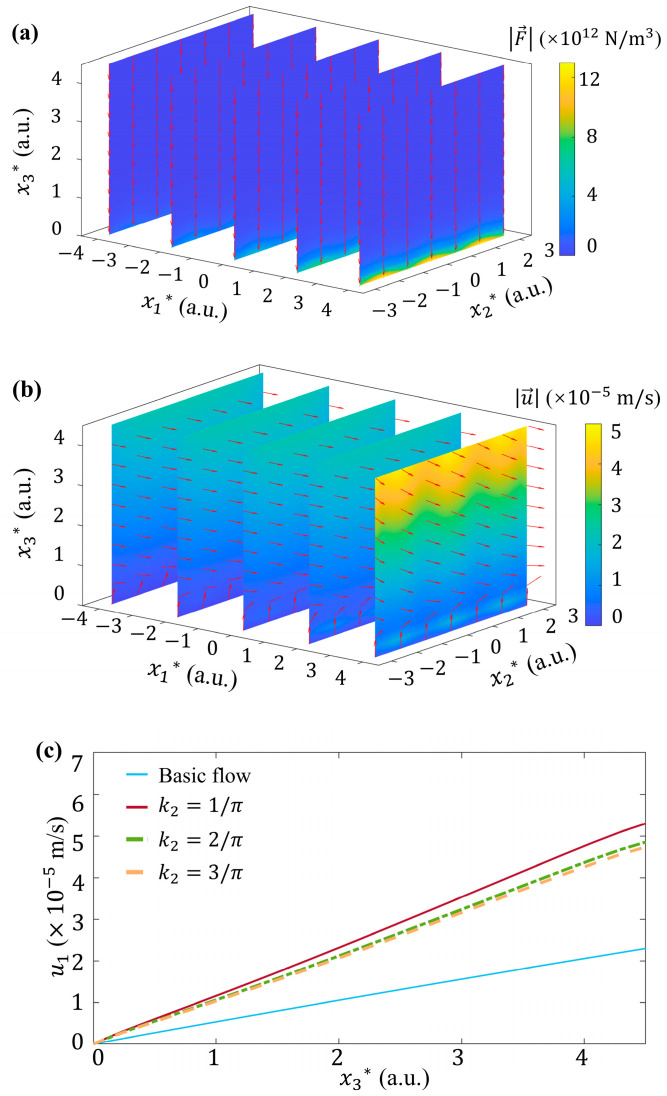
Influence of $k_{2}$ on the flow, with k=222 1/m, $A^{*}=0.1$, and $\zeta_{0}=-36$ mV. 3D distribution of (**a**) electric volume force $\vec{F_{e}}$ and (**b**) fluid velocity $\vec{u}$ with wavenumber $k_{2}=2/\pi$. (**c**) Plot of streamwise velocity component, $u_{1}$, versus dimensional height ${x_{3}}^{*}$ at different $k_{2}$, where ${x_{1}}^{*}=4$ and ${x_{2}}^{*}=0$.

At last, we discuss the influence of spanwise distributions of *ζ* potential on the flow, under k=222 1/m, $A^{*}=0.1$, and $\zeta_{0}=-36$ mV. Larger $k_{2}$ indicates a wavier distribution of *ζ* potential in the $x_{2}$ direction, which induces a stronger gradient of *ζ* potential and more peak value positions of $\vec{F_{e}}$ as well. At $k_{2}=2/\pi$ as an example, the electric volume force $\vec{F_{e}}$ and the corresponding fluid velocity $\vec{u}$ both exhibit wavy distribution along the spanwise direction, as shown in Figure 12a,b. Even if the peak values of $\left|\vec{F_{e}}\right|$ at $k_{2}=2/\pi$ are equal to that of $k_{2}=1/\pi$ shown in Figure 8b, the peak value of $\left|\vec{u}\right|$ becomes decreased a little bit relative to Figure 9a. The increasing $k_{2}$ also decreases the streamwise velocity $u_{1}$ and its gradient $\partial u_{1}/\partial x_{3}$ (Figure 12c). Therefore, a smaller τ of flow can be concluded.

All in all, it can be seen, when taking the influence of 2D *ζ* potential into account, the wall shear stress can be significantly enhanced. For example, at k=222 1/m, $A^{*}=0.1$, $\zeta_{0}=-36$ mV, and $k_{2}=1/\pi$, the maximum τ can be up to 1.074 Pa, which is about 2.5 folds larger than the basic flow. A stronger wall shear stress indicates a stronger interaction between wall and flow. This could be important in many shear-sensitive circumstances, especially on biological materials. One example is that the flow shear stress on cells and tissues may be larger than previously expected. Studying the different control parameters of 2D potential distribution can help us better understand the effect of charge distribution on interfacial flow, and according to Equation (16), we can even realize of adjustment of wall shear stress by changing these parameters.

### 5.5. Influence of Other Parameters

In addition to the parameters of the zeta potential distribution discussed above, there are also several other parameters that affect the EK flow at the solid–liquid interface, for example, the electric conductivity and viscosity of the fluid. On the one hand, the electric conductivity is closely related to Debye length, which affects the distribution of electric potential φ and charge density $\rho_{e}$ in the EDL. This in turn affects electric volume force $\vec{F_{e}}$ and the flow as delineated in Equations (2)–(5). On the other hand, the influence of viscosity is also important. The EK flow induced by non-uniform *ζ* potential is intrinsically a balance between EVF and viscosity [46,47], primarily due to the balance of electric volume force and the viscous effect. The changing of viscosity could affect both the velocity profile and wall shear stress τ near the wall. However, a comprehensive exploration on these parameters accompanied with zeta potential variation are beyond the scope of this manuscript and will be studied in future works.

## 6. Conclusions

In this investigation, the influence of the non-uniform 2D distribution of *ζ* potential on the flow at the solid–liquid interface has been investigated numerically. Since the electric volume force $\vec{F_{e}}$ can be rotational, it further drives the fluid to produce a swirling flow. The streamwise vortices, which are important in the turbulent boundary layer, are theoretically predicted in this laminar flow model when considering the 2D distribution of *ζ* potential. This observation implies it is necessary to reconsider the origin of streamwise vortices in turbulent boundary layer, from the perspective of electrokinetic flow. In addition, when considering the influence of *ζ* potential distribution, the wall shear stress can be apparently larger relative to no *ζ* potential considered. This is important for shear-sensitivity circumstances, particularly in biomedical and medical devices, and in vivo. We hope the present work provides a theoretical foundation to understand the complex development and evolution mechanism through various electrical and dynamics parameters, and provides a new way to control the vortices and shear stress in boundary layers.

## Figures and Tables

**Table 1:** Properties of the material.

Material	$\rho_{f}$ $(kg/m^{3}$)	μ $(\times 10^{-5}\;Pa\cdot s$)	$\varepsilon_{r}$
Liquid water	997	89.57	80

**Table 2:** Detailed information of the grid in the model.

Parameter	Value
Number of grid elements	7,106,000
Number of grid vertices	7,275,576
Minimum grid element quality	1.0
Average grid element quality	1.0
Grid element volume ratio	$3.427\times 10^{-5}$
Grid volume	20 μm^3^
$\mathrm{Minimum}\;\mathrm{grid}\;\mathrm{size}\;\mathrm{in}\;x_{1}$ direction	6.68 nm
$\mathrm{Maximum}\;\mathrm{grid}\;\mathrm{size}\;\mathrm{in}\;x_{1}$ direction	20 nm
$\mathrm{Minimum}\;\mathrm{grid}\;\mathrm{size}\;\mathrm{in}\;x_{2}$ direction	4.42 nm
$\mathrm{Maximum}\;\mathrm{grid}\;\mathrm{size}\;\mathrm{in}\;x_{2}$ direction	13.26 nm
$\mathrm{Minimum}\;\mathrm{grid}\;\mathrm{size}\;\mathrm{in}\;x_{3}$ direction	1.33 nm
$\mathrm{Maximum}\;\mathrm{grid}\;\mathrm{size}\;\mathrm{in}\;x_{3}$ direction	180 nm

## Data Availability

The original contributions presented in the study are included in the article, further inquiries can be directed to the corresponding author.
